# Evolution of myocardial oedema and fibrosis in HIV infected persons after the initiation of antiretroviral therapy: a prospective cardiovascular magnetic resonance study

**DOI:** 10.1186/s12968-022-00901-0

**Published:** 2022-12-19

**Authors:** Pieter-Paul S. Robbertse, Anton F. Doubell, Carl J. Lombard, Mohammed A. Talle, Philip G. Herbst

**Affiliations:** 1 Division of Cardiology, Department of Medicine, Faculty of Medicine and Health Sciences, Stellenbosch University and Tygerberg Hospital, Cape Town, South Africa; 2 University of Pittsburgh HIV-Comorbidities Research Training Programme in South Africa, Cape Town, South Africa; 3 Biostatistics Unit, South African Medical Research Council, Cape Town, South Africa; 4 Division of Epidemiology and Biostatistics, Department of Global Health, Stellenbosch University, Cape Town, South Africa; 5 Department of Medicine, Faculty of Clinical Sciences, College of Medical Sciences, University of Maiduguri, Maiduguri, Nigeria

**Keywords:** HIV-associated cardiovascular disease, HIV-associated cardiomyopathy, Subclinical cardiovascular disease, Myocardial inflammation, Myocardial fibrosis, Parametric mapping, Late gadolinium enhancement, Antiretroviral therapy, Prospective, Low- and middle income countries

## Abstract

**Background:**

Human immunodeficiency virus (HIV) infected persons on antiretroviral therapy (ART) have been shown to have functionally and structurally altered ventricles and may be related to cardiovascular inflammation. Mounting evidence suggests that the myocardium of HIV infected individuals may be abnormal before ART is initiated and may represent subclinical HIV-associated cardiomyopathy (HIVAC). The influence of ART on subclinical HIVAC is not known.

**Methods:**

Newly diagnosed, ART naïve persons with HIV infection were enrolled along with HIV uninfected, age- and sex-matched controls. All participants underwent comprehensive cardiovascular assessment, including contrasted cardiovascular magnetic resonance (CMR) with multiparametric mapping on a 1.5T CMR system. The HIV group was started on ART (tenofovir/lamivudine/dolutegravir) and prospectively evaluated 9 months later. Cardiac tissue characterisation was compared in, and between groups using the appropriate statistical tests for the cross sectional data and the paired, prospective data respectively.

**Results:**

Seventy-three ART naïve HIV infected individuals (32 ± 7 years, 45% female) and 22 healthy non-HIV subjects (33 ± 7 years, 50% female) were enrolled. Compared with non-HIV healthy subjects, the global native T1 (1008 ± 31 ms vs 1032 ± 44 ms, p = 0.02), global T2 (46 ± 2 vs 48 ± 3 ms, p = 0.006), and the prevalence of pericardial effusion (18% vs 67%, p < 0.001) were significantly higher in the HIV infected group at diagnosis. Global native T1 (1032 ± 44 to 1014 ± 34 ms, p < 0.001) and extracellular volume (ECV) (26 ± 4% to 25 ± 3%, p = 0.001) decreased significantly after 9 months on ART and were significantly associated with a decrease in the HIV viral load, decreased high sensitivity C-reactive protein, and improvement in the CD4 count (p < 0.001). Replacement fibrosis was significantly higher in the HIV infected group than controls (49% vs 10%, p = 0.02). The prevalence of late gadolinium enhancement did not change significantly over the 9-month study period (49% vs 55%, p = 0.4).

**Conclusion:**

Subclinical HIVAC may already be present at the time of HIV diagnosis, as suggested by the combination of subclinical myocardial oedema and fibrosis found to be present *before* administration of ART. Markers of myocardial oedema on tissue characterization improved on ART in the short term, however, it is unclear if the underlying pathological mechanism is halted, or merely slowed by ART. Mid- to long term prospective studies are needed to evaluate subtle myocardial changes over time and to assess the significance of subclinical myocardial fibrosis.

## Background

Human immunodeficiency virus (HIV) infection is associated with cardiovascular dysfunction and mortality [1]. Since the widespread roll-out of antiretroviral therapy (ART), cardiovascular disease (CVD) has become less frequent and HIV has evolved into a chronic disease [2].

Despite the advances in ART, people living with HIV remain at higher risk for CVD than the general population [3] and it is likely that pathological processes in the cardiovascular system are only partially addressed by ART. A spectrum of CVD is observed in people living with HIV and the disease profiles differ between persons on ART and those not on ART [4–7]. In contrast to what is seen in high income countries﻿, where coronary artery disease is highly prevalent, HIV-associated cardiomyopathy (HIVAC) remains a significant contributor to myocardial disease in sub-Saharan Africa [4, 8, 9].

There is mounting evidence to suggest that the hearts of HIV infected individuals may already be functionally and structurally abnormal at the time of HIV diagnosis with different possible substrates for cardiac pathology [10–13]. In the presence of appropriate driving factors, these subclinical abnormalities may represent an early stage of myocardial pathology on a continuum toward dilated cardiomyopathy and irreversible structural change [5]. The mechanisms responsible for these abnormalities remain incompletely understood. It is postulated that several infection-, patient- and environmental aetiologies are at play, resulting in inflammation as a common end-point [13–19].

Cardiovascular magnetic resonance imaging (CMR) is a powerful diagnostic modality for assessing both structural and functional cardiovascular parameters. In addition, CMR’s tissue characterisation ability is of particular interest to assess for the presence and evolution of myocardial oedema, inflammation, and fibrosis in the HIV population, particularly given the prevailing inflammatory hypothesis. The use of novel multiparametric mapping techniques have created the opportunity to establish quantitative imaging biomarkers to aid with diagnostics and prospectively study of these patients [20].

We hypothesise that HIVAC is already demonstrable at the time of HIV diagnosis as subclinical myocardial tissue abnormalities. Based on the prevailing inflammatory hypothesis, we further predict that tissue abnormalities will reverse, at least in part, on ART. Using CMR, we set out to prospectively evaluate the hearts of a contemporary HIV infected cohort before and 9 months after initiation of ART.

## Methods

### Study design and participants

Detailed methodology regarding the recruitment process of the project have been published elsewhere [21] and this cohort has subsequently been expanded in this study. In short: Newly diagnosed, HIV infected individuals were recruited into a prospective cohort study in the Western Cape, South Africa. Participants were recruited as out-patients as they tested positive for HIV, along with a healthy, HIV uninfected control group from the same areas, frequency matched for age and sex. The HIV status of all persons were confirmed with a serum HIV enzyme-linked immunosorbent assay and eligible participants were enrolled before the initiation on first line ART (tenofovir/lamivudine/dolutegravir). The ART naïve status of participants was cross checked on a centralised pharmacy system. All participants recruited after March 2020 underwent severe acute respiratory syndrome coronavirus-2 polyumerace chain reaction (PCR) testing before inclusion. All patients aged 18 to 55 years, without known current or prior cardiac disease and without ART use prior to enrolment, who were not pregnant or acutely unwell (including current coronavirus disease-19), were deemed eligible for inclusion. Potential participants with a contraindication to CMR or gadolinium contrast, were excluded. Participants diagnosed with tuberculosis (TB) coinfection, were not excluded. HIV/TB coinfected participants were enrolled at least 2 weeks after initiation on TB treatment while still ART naïve. All treatment and workup provided to HIV infected participants were in line with South African treatment guidelines [22].

### Clinical data collection and follow up

Data were collected at three time points for HIV infected participants: A baseline visit prior to ART, an interim visit at 4 months, and a final follow-up after at least 9 months on ART. The HIV uninfected healthy subjects had a once-off research visit. Full evaluation including anthropometric, biochemical, immunological, virological, electrocardiogram (ECG), and CMR investigations were performed at baseline and final follow up. Additional CD4 count and HIV viral load measurements were made at the interim visit. Participants were seen in the morning following a fast of at least 10 h. The World Health Organisation (WHO) HIV clinical stage was documented [23]. Blood pressure was measured in the seated position after a 10-min rest on the same wall-mounted sphygmomanometer unit (Welch Allyn, New York, USA). Mean arterial pressure was calculated as [(1/3 × pulse pressure) + diastolic blood pressure] [24]. Height and weight were measured, and body mass index (BMI) was calculated. Waist circumference was measured standing at the level of the umbilicus. A 12-lead ECG was performed. A 6 min walk test (6MWT) was recorded using standardised methodology [25]. Fasting blood samples were analysed by the on-site National Health Laboratory Service for measurement of urea and electrolytes, glucose, blood lipids, full blood count, differential cell count, HIV enzyme-linked immunosorbent assay, high sensitivity C-reactive protein (hsCRP), flow cytometry based CD4- and CD8 count, and HIV-1 viral load. The linear range for the measurement of HIV viral load by our core laboratory is 20 to 10,000,000 copies/ml or 1.3 to 7 log (Abbot Alinity M HIV-1 assay, Abbott Park, Illinois, USA). We defined persistent viraemia as a viral load of > 200 copies/ml in persons on ART at interim follow-up or at 9 months in accordance with contemporary research [26]. Gender-specific estimated glomerular filtration rate (eGFR) was calculated using the Cockcroft–Gault formula [22]. At the final visit, severe acute respiratory syndrome coronavirus 2 (SARS-CoV-2) immunoglobulin G antibodies were tested in the HIV group.

### Cardiovascular magnetic resonance

#### Image acquisition

CMR studies were performed on a single 1.5T CMR system (Magnetom Avanto, Siemens Healthineers, Erlangen, Germany) with commercially available cardiac sequences. Baseline and follow up studies were performed by the same operator using standard methods and imaging planes [27]. Breath-held, ECG gated, balanced steady-state free precession (bSSFP) cine images were obtained for the assessment of cardiac mass, volumes, function, and morphological evaluation. Additional breath-held images were acquired to evaluate myocardial tissue characteristics. These included 8 mm T1-weighted turbo spin echo images, T2 short-tau inversion recovery (STIR) images, native and post-contrast T1 mapping, T2 mapping, segmented early gadolinium enhancement (EGE) images, and late gadolinium enhancement (LGE) images. Images were acquired in four-, three-, and two chamber orientations as well as basal, mid, and apical slices in short axis. Short axis stacks covering the left ventricle (LV) from base to apex with no gap were performed for EGE and LGE images. For native T1 mapping, a pre-contrast 3(3)3(3)5 modified-Look Locker inversion recovery (MOLLI) sequence was employed [28]. T2 maps were acquired using bSSFP. Single shot and segmented bSSFP EGE images were acquired 1–2 min after injection of 0.2 ml/kg of a macrocyclic gadolinium-based contrast agent. High resolution magnitude reconstruction and phase sensitive inversion recovery LGE images were acquired 10–12 min after the administration of the contrast agent. Optimal inversion time was guided by a scout sequence and visually confirmed by the operator. Where necessary, phase swapping or specific cross-cuts were acquired to assess areas with LGE. Post-contrast T1 maps were acquired at 15–16 min after contrast injection, using a post-contrast MOLLI sequence.

#### Image analysis

PSR performed unblinded post processing and analysis on all the CMR data using cvi^42^ (version 5.11.2, Circle Cardiovascular Imaging, Calgary, Alberta, Canada). Semi-automated, artificial intelligence assisted endocardial and epicardial LV borders were traced at end-systole and end-diastole to determine the LV volume and mass. Papillary muscles were excluded from the LV blood pool and contributed to the LV mass. Endocardial borders for the right ventricle (RV) were drawn in a similar manner to acquire RV volumes. For quantitative multiparametric mapping measurements, the LV endocardial and epicardial borders were traced in basal, mid, and apical short axis sections to acquire mean values on STIR images, EGE images, native T1-, T2- and extracellular volume fraction (ECV) maps. Short axis ECV maps were created using post processing software.

The blood pool contour was drawn in native and post-contrast T1 maps, avoiding papillary muscle and trabeculations. To increase accuracy and reproducibility, conservative contouring was performed to avoid the blood-myocardial interface and epicardial fat. In addition, a 20% epicardial- and a 10% endocardial offset were employed during post processing of mapping parameters. To calculate the myocardial: skeletal signal intensity ratio (SIR) on STIR and EGE images, a region of interest (ROI) was drawn over the serratus anterior muscle in the same slice [29]. Qualitative analyses were performed on LGE images. LGE had to fulfill predetermined study criteria to be deemed present: Signal in the late phase was excluded if it was interpreted to be blood pool or extracardiac fat. This was determined by cross-checking with cine images and other pulse sequences evaluating the same region. LGE was reported as positive if visualised in 2 orthogonal views or 2 adjacent slices to mitigate the misinterpretation of artefact as LGE.

MAT re-read 15 cases in a random and blinded fashion to assess the inter-reader variability within the study. PGH supervised the CMR analysis and reviewed key findings and measurements. He was blinded to the clinical information. PSR and MAT both have 3 years and PGH has 7 years’ experience in formal CMR analysis.

### Statistical analysis

Sample size calculation was performed for CMR-measured LV volumes, mass, and ejection fraction (LVEF) using paired samples t-test for the prospective evaluation of the HIV infected group. Statistical analyses were performed using SPSS (version 27, Statistical Package for the Social Sciences, International Business Machines, Inc., Armonk, New York, USA) and STATA (version 17.0, Stata Corporation, College Station, Texas, USA). Continuous data are presented as mean ± standard deviation. For non-normally distributed data, the median and interquartile ranges are stated. Categorical data is presented as frequencies with percentages. The normality of data was assessed using the Shapiro–Wilk test. For cross sectional analyses between the HIV infected and control group, the Chi-square test, independent samples t-test, and Mann–Whitney U tests were used as appropriate. For prospective analyses of the HIV infected group, the paired samples t-test, Wilcoxon signed-rank test, or McNemar test was employed as appropriate. Inter-reader reliability on quantitative data was assessed using interclass correlation coefficient (ICC). A two-way random, average measurements with absolute agreement model was used. Cohen’s Kappa was used to evaluate the agreement of categorical variables between readers. Bivariate correlations were calculated using Spearman’s Rho. Multivariate analysis with native T1 as outcome variable was performed with quantile regression models. Statistical significance was 2-tailed and defined as a p-value ≤ 0.05.

## Results

### Study group characteristics

**Fig. 1:**
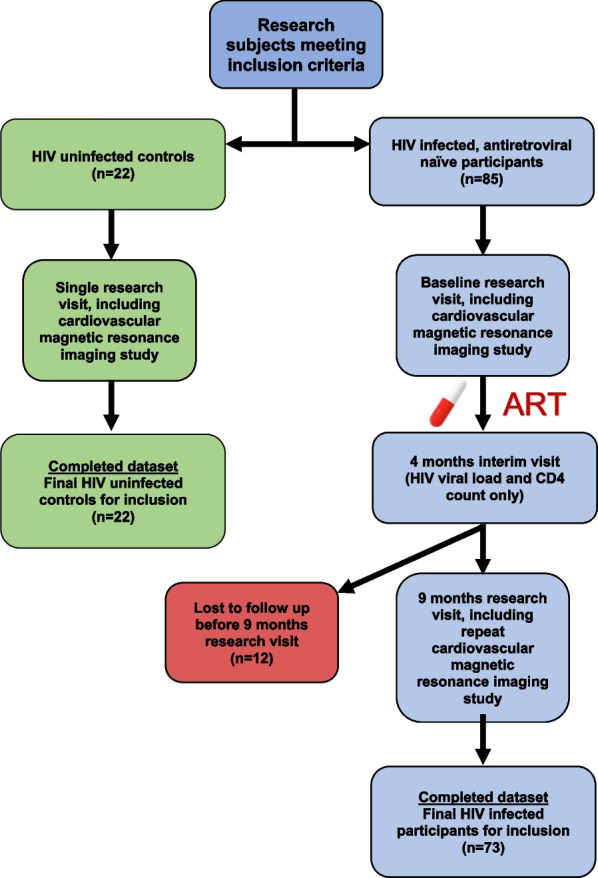
Study flow chart. *ART* antiretroviral therapy

All participants that underwent a 9-months follow up CMR were included in the study (Fig. 1). The study group’s demographics and clinical data are shown in Table 1. The HIV infected group and healthy controls were well matched in terms of age and sex. Both groups were relatively young with a mean age of 33 and 32 years, respectively. The income of the HIV infected group was considerably lower compared with controls, and reflect the lower socio-economic status of this population. Although the HIV infected group had more smokers (49% vs 27% in the control group), this did not reach statistical significance. Ethanol use showed a wide spread in the data, but proved statistically similar in the naïve group and controls. 97% of the cohort a dolutegravir-based regimen. After initiation on ART, participants had the tendency to consume considerably less alcohol. Chronic disease and medication use were rare, and comparable between the controls and HIV infected group. Two HIV infected participants had a known diagnosis of hypertension. During follow up, one HIV infected participant developed hypertension and received treatment. Metabolic syndrome was rare with only one control and two participants fulfilling the National Cholesterol Education Program Adult Treatment Panel III [30] criteria at enrolment.

**Table 1:** Study population

Parameter	Control group	HIV infected ART naïve (n = 73) (Naïve)	p value^a^	HIV infected 9 months on ART	p value^b^
		ART naïve			
	(n = 22)	(n = 73)		(n = 73)	
	(Controls)	(Naïve)		(ART)	
Age (years)	33 ± 7	32 ± 7	0.6	–	–
Female sex	11 (50%)	33 (45%)	0.6	–	–
Monthly household income (South African rand per month)					
< 5000	4 (18%)	53 (73%)	**0.02**	–	–
5000 to 20,000	14 (77%)	19 (26%)			
> 20,000	4 (18%)	1 (1%)			
Smoking history	6 (27%)	36 (49%)	0.07	37 (51%)	
Ethanol (units per week)	0.5 (0 to 7)	4 (0 to 12)	0.2	0 (0 to 11)	**0.01**
Waist circumference (cm)	95 ± 18	80 ± 9	**< 0.001**	82 ± 10	0.06
Body mass index (kg/m^2^)	30 ± 8	23 ± 4	**< 0.001**	24 ± 4	**< 0.001**
World Health Organisation HIV clinical stage					
I	–	28 (38%)	–	28 (38%)	–
II		19 (26%)		19 (26%)	
III		25 (34%)		24 (34%)	
IV		1 (1%)		2 (1%)	
On treatment for TB		10 (14%)	–	2* (3%)	–
Pulmonary	–	8		0	
Extra-pulmonary		2		2	
Days since HIV diagnosis	–	8 (5 to 26)	-	300 (283 to 389)	–
Time to interim follow up (months)	–	–	–	4 (3 to 5)	–
Time to 9 months follow up (months)	–	–	–	9 (9 to 13)	–
History of proven COVID-19 (mild disease)	1 (5%)	None		1 (1%)	–
Antibody evidence of SARS-CoV-2 infection in unimmunised persons	None	Not performed at baseline	–	18 (25%)	–
Medications					
Salbutamol metered dose inhaler	1 (5%)	1 (1%)	–	1 (1%)	–
Rifampicin/isoniazid/pyrazinamide/ethambutol	–	10 (14%)		2 (3%)	
Trimethoprim/sulphamethoxazole	–	21 (29%)		14 (19%)	
Isoniazid prophylaxis	–	1 (1%)		43 (59%)	
Pyridoxine	–	10 (14%)		43 (59%)	
Tenofovir/lamivudine/dolutegravir	–	Naïve		71 (97%)	
Losartan	–	–		2 (3%)	
Amlodipine/hydrochlorothiazide	–	1 (2%)		3 (4%)	
Statins	–	–		1 (1%)	
Clinical course on ART					
Immunological failure^**^ at interim or final follow up	–	–	–	15 (21%)	–
Viral load > 200 copies/ml at interim or 9 months	–	–	–	15 (21%)	–
6 min walk test distance (m)	637 ± 84	618 ± 92	0.4	621 ± 75	0.7

Bold values indicate *p* value of ≤ 0.05

Continuous variables are mean ± standard deviation or median (interquartile range) unless otherwise specified

*HIV* Human immunodeficiency virus, *TB* Tuberculous disease, *COVID-19* Coronavirus disease-19, *SARS-CoV 2* Severe acute respiratory syndrome coronavirus-2, *ART* Antiretroviral therapy

^a^Controls vs naïve

^b^Naïve vs ART

^*^One additional patient developed tuberculosis after recruitment

^**^Failure was defined as a CD4 drop to below baseline or a 50% decrease from the on-treatment peak value

The median time from enrolment to repeat CMR evaluation was 9 months (Interquartile range: 9 to 13 months).

### The general health of the HIV infected group

Sixty six percent of the cohort had early (WHO I or stage II) disease. A third of the participants visited their health centre due to significant, unexplained weight loss and were otherwise well. The remainder of the HIV group presented due to contact tracing or with HIV related complications. These included dermatological manifestations of HIV, lymphadenopathy, respiratory-, gastrointestinal-, and miscellaneous complaints. HIV/TB coinfection was found to be prevalent among newly diagnosed people living with HIV. Ten naïve participants (14% of the HIV group) had TB disease diagnosed around the time of enrolment. All but one of the TB cases were mild and managed on an out-patient basis. Extrapulmonary TB was diagnosed incidentally in 2 cases at baseline (abdominal and pleural TB respectively). The 6MWT demonstrated a comparable functional status between the naïve group and controls (618 vs 637 m, p = 0.4). No HIV infected participant reported any significant loss of functional capacity or dyspnoea. Furthermore, ART did not cause a significant improvement in the 6MWT.

Biochemical and immunological data are shown in Table 2*.* At 9 months on ART, participants had a median viral load of 20 copies/ml (Interquartile range: 20 to 36). Median hsCRP measured 50% higher in the naïve group than controls (3.6 vs 2.4 mg/l). This did however not reach statistical significance (p = 0.5). A marked decrease in the median hsCRP was observed when comparing the HIV infected participants at enrolment and 9-month follow-up (3.4 to 1.9 mg/l, p = 0.01).

**Table 2:** Biochemical and immunological evaluation

Parameter	Control group	HIV infected ART naïve	p value^a^	HIV infected 9 months on ART	p value^b^
		ART naïve			
	(n = 22)	(n = 73)		(n = 73)	
	(Controls)	(Naïve)		(ART)	
Serum creatinine (μmol/l)	72 ± 14	70 ± 14	0.6	82 ± 16	**< 0.001**
Estimated glomerular filtration rate (ml/min/1.73 m^2^)	111 ± 38	92 ± 22	**0.03**	83 ± 19	**< 0.001**
Fasting blood glucose (mmol/l)	5.0 ± 0.6	4.5 ± 0.6	**< 0.001**	4.7 ± 0.6	**< 0.001**
Haematocrit (%)	42 ± 4	38 ± 6	**0.03**	41 ± 5	**< 0.001**
Total fasting serum cholesterol (mmol/l)	4.3 (3.9 to 4.9)	3.4 (2.9 to 4.0)	**< 0.001**	3.5 (3.0 to 4.1)	0.1
Triglycerides (mmol/l)	0.9 (0.6 to 1.2)	0.8 (0.7 tot 1.2)	0.8	0.7 (0.6 to 1.2)	**0.03**
HDL cholesterol (mmol/l)	1.4 (1.2 to 1.5)	1.0 (0.8 to 1.3)	**0.001**	1.2 (1.0 to 1.4)	**< 0.001**
LDL cholesterol (mmol/l)	2.7 (2.0 to 3.1)	1.8 (1.5 to 2.4)	**0.004**	1.9 (1.6 to 2.4)	0.9
High sensitivity C-reactive protein (mg/l)	2.4 (1.1 to 9.2)	3.4 (0.8 to 13.7)	0.5	1.9 (0.5 to 7.5)	**0.01**
CD4 count (cells/μl)	–	289 (170 to 410)	–	378 (271 to 593)	**< 0.001**
CD8 count (cells/μl)	–	817 (584 to 1147)	–	624 (446 to 858)	**< 0.001**
CD4:CD8 ratio	–	0.35 (0.19 to 0.45)	–	0.60 (0.39 to 0.94)	**< 0.001**
HIV viral load (copies/ml)		78,279 (9796 to 336,900)	–	20* (20 to 36)	**< 0.001**
HIV viral load (log copies/ml)	–	4.9 (4.0 to 5.5)	–	1.3* (1.3 to 1.6)	**< 0.001**

Bold values indicate *p* value of ≤ 0.05

Continuous variables are mean ± standard deviation or median (interquartile range) unless otherwise specified

*HDL* high density lipoprotein, *LDL* low density lipoprotein, *HIV* Human immunodeficiency virus

^a^Controls vs naïve

^b^Naïve vs ART

^*^20 copies/ml (log = 1.3) is the lower limit of measurement for our HIV-1 assay

### Pericardial effusions

CMR data are shown in Table 3. The presence of small (< 10 mm), haemodynamically insignificant pericardial effusions were highly prevalent in the naïve group compared with controls (67% vs 18%, p < 0.001). No study participant had pericardial LGE. The pericardial effusions were observed in the absence of pericardial thickening and were not associated with the presence of TB disease.

**Table 3:** Cardiovascular magnetic resonance and tissue characterisation parameters

Parameter	Control group	HIV infected	p value^a^	HIV infected 9 months on ART	p value^b^
		ART naïve			
	(n = 22)	(n = 73)		(n = 73)	
	(Controls)	(Naïve)		(ART)	
LV mass indexed to height (g/m)	60 (51 to 71)	63 (53 to 73)	**0.05**^**#**^	63 (53 to 75)	0.8
LV end diastolic volume indexed to height (ml/m)	81 ± 13	86 ± 16	**0.03**^**##**^	89 ± 15	**0.007**
LV sphericity Index	0.53 ± 0.04	0.53 ± 0.05	0.6	0.52 ± 0.04	0.4
LV ejection fraction (%)	63 ± 5	60 ± 6	**0.03**	59 ± 5	0.6
Presence of pericardial effusion	4 (18%)	49 (67%)	**< 0.001**	46 (63%)	0.6
Abnormal T2 SIR region of interest	2.0 ± 0.2	2.0 ± 0.2	1.0	2.1 ± 0.4	**0.05**
Basal T2 SIR	1.5 ± 0.3	1.5 ± 0.2	0.7	1.6 ± 0.2	0.08
Midventricular T2 SIR	1.5 ± 0.2	1.5 ± 0.2	0.9	1.5 ± 0.2	0.5
Apical T2 SIR	1.4 ± 0.2	1.5 ± 0.2	0.3	1.5 ± 0.2	0.9
Global T2 SIR	1.5 ± 0.2	1.5 ± 0.2	0.5	1.5 ± 0.2	0.4
Basal native T1 mapping (ms)	1004 ± 24	1027 ± 38	**0.01**	1008 ± 32	**< 0.001**
Midventricular native T1 mapping (ms)	1003 ± 31	1025 ± 42	**0.02**	1011 ± 34	**0.008**
Apical native T1 mapping (ms)	1016 ± 43	1044 ± 61	**0.05**	1024 ± 42	**0.001**
Global native T1 mapping (ms)	1008 ± 31	1032 ± 44	**0.02**	1014 ± 34	**< 0.001**
Basal T2 mapping (ms)	45 ± 2	47 ± 3	**0.002**	47 ± 2	0.2
Midventricular T2 mapping (ms)	46 ± 2	48 ± 3	**< 0.001**	48 ± 2	0.4
Apical T2 mapping (ms)	48 ± 2	49 ± 3	0.07	50 ± 2	0.7
Global T2 mapping (ms)	4.6 ± 2	48 ± 3	**0.006**	48 ± 2	0.5
Basal EGE SIR	3.0 (2.3 to 3.6)*	3.0 (2.6 to 4.5) **	0.2	3.2 (2.6 to 4.1)***	0.9
Midventricular EGE SIR	2.6 (2.2 to 4.1)*	3.0 (2.5 to 4.0)**	0.4	3.0 (2.5 to 4.0)***	1.0
Apical EGE SIR	2.8 (2.2 to 3.8)*	2.9 (2.5 to 3.9)**	0.7	2.9 (2.4 to 4.1)***	0.9
Global EGE SIR	2.7 (2.3 to 3.9)*	2.9 (2.5 to 4.2)**	0.3	3.1 (2.5 to 4.0)***	0.8
Basal ECV (%)	23 ± 2*	25 ± 4	0.1	24 ± 3	**0.003**
Midventricular ECV (%)	24 ± 3*	25 ± 4	0.2	25 ± 3	**0.03**
Apical ECV (%)	25 ± 4*	28 ± 5	0.09	26 ± 3	**< 0.001**
Global ECV (%)	24 ± 38*	26 ± 4	0.1	25 ± 3	**0.001**
LGE present	1 (10%)*	35 (49%)	**0.02**	40 (55%)	0.2
Pattern of LGE					
Subepicardial	–	18 (50%)	–	18 (45%)	0.7
Midmyocardial	1 (100%)^*^	12 (33%)		17 (43%)	
Mixed	–	6 (17%)		5 (12%)	
Segments with LGE					
Basal infero-posterior	–	33 (49%)	–	35 (53%)	0.7
Mid infero-posterior	1 (100%)^*^	13 (19%)		10 (15%)	
Basal septum	–	5 (7%)		8 (12%)	
Mid septum	–	2 (3%)		2 (3%)	
Basal antero-lateral	–	8 (12%)		6 (9%)	
Mid antero-lateral	–	2 (3%)		2 (3%)	
Apical segments	–	4 (6%)		3 (5%)	

Bold values indicate *p* value of ≤ 0.05

Continuous variables are mean ± standard deviation or median (interquartile range) unless otherwise specified

*ECV* extracellular volume fraction, *EGE* early gadolinium enhancement, *LGE* late gadolinium enhancement, *LV* left ventricle/left ventricular, *SIR* signal intensity ratio, *STIR* short tau inversion recovery

^*^50% of the controls received gadolinium contrast (n = 11)

^**^Three outliers were excluded from analysis

^***^One outlier was excluded from analysis

^#^Mean difference = 6 g when corrected for age, sex, race, estimated glomerular filtration rate, and current tuberculous disease

^##^Mean difference = 6 ml when corrected for age, sex, race, ethanol use, and current tuberculous disease

### STIR and EGE imaging

Although a trend of abnormality was observed, global T2 SIR did not show a statistical difference between the naïve group and controls (p = 0.5). When evaluating the HIV group over time, a trend of increasing T2 SIR, most notable at the basal segments, was observed. This however did not reach our study’s requirements for significance (p = 0.08). EGE SIR did not demonstrate any difference amongst the study groups.

### Multiparametric mapping

**Fig. 2:**
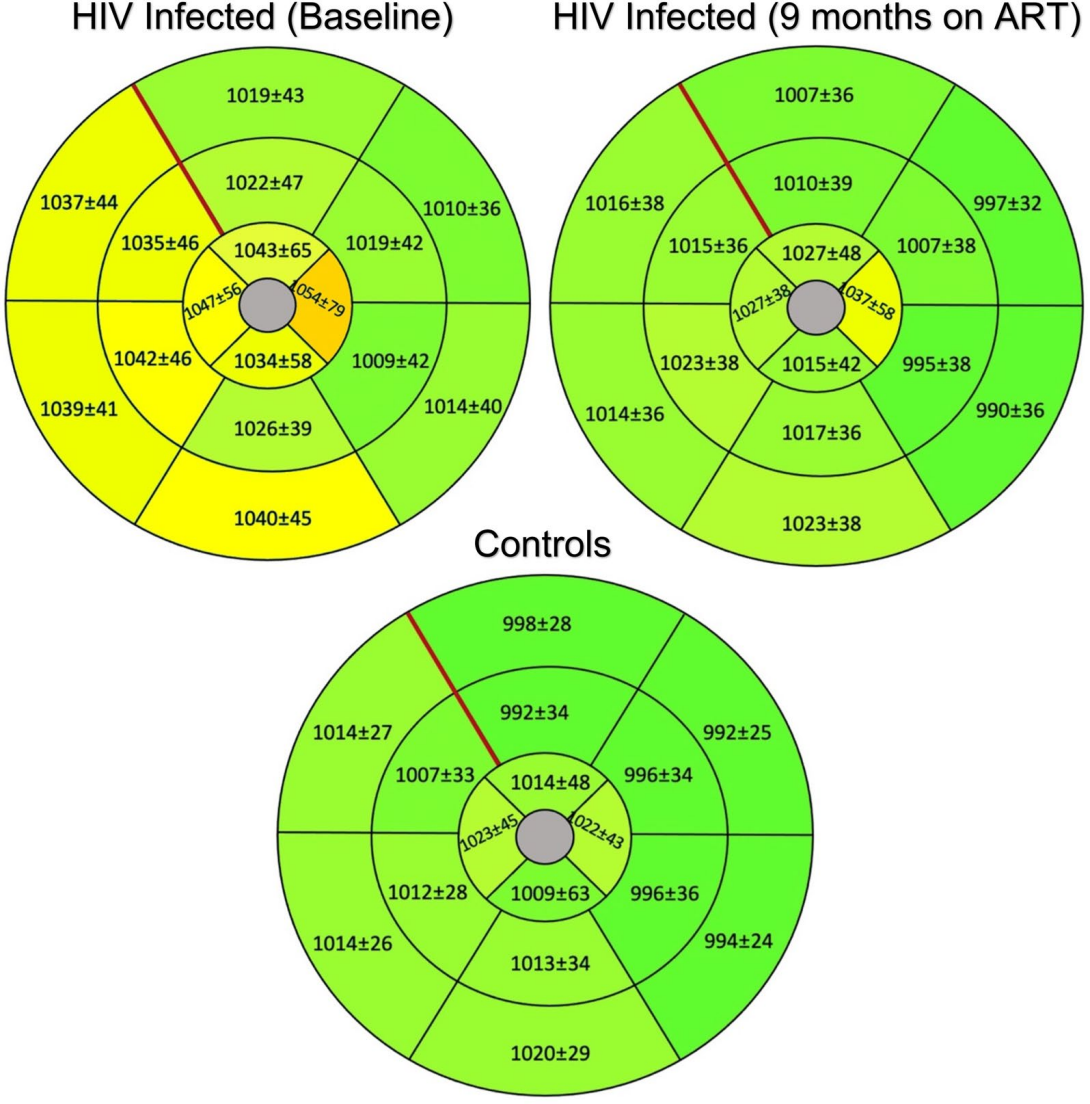
Polar maps of the study groups demonstrating segmented, mean native T1 in milliseconds. Compared with the control group (n = 22), the native T1 of the human immunodeficiency virus (HIV) infected group at baseline (n = 73) is elevated in all segments. After 9 months of antiretroviral treatment (ART), the native T1 decreased in all segments of HIV-infected participants. The red line indicates the anterior right ventricular insertion site

The segmental native T1 times of the study groups are shown in Fig. 2. Both the global native T1 and T2 mapping values were higher in the naïve group compared with controls. These differences were most notable at the heart base with mean differences of 23 ms (ms) and 2 ms for global native T1 and T2 respectively. The mean global ECV of the control group measured 2% lower compared with the naïve group. Only a small number of controls received contrast, and this did not reach statistical significance when compared to the ART naïve group (p = 0.1). However, global native T1 (obtained in all controls), demonstrated a high linear correlation with ECV across all study groups (r_s_ = 0.8, p < 0.001).

**Fig. 3:**
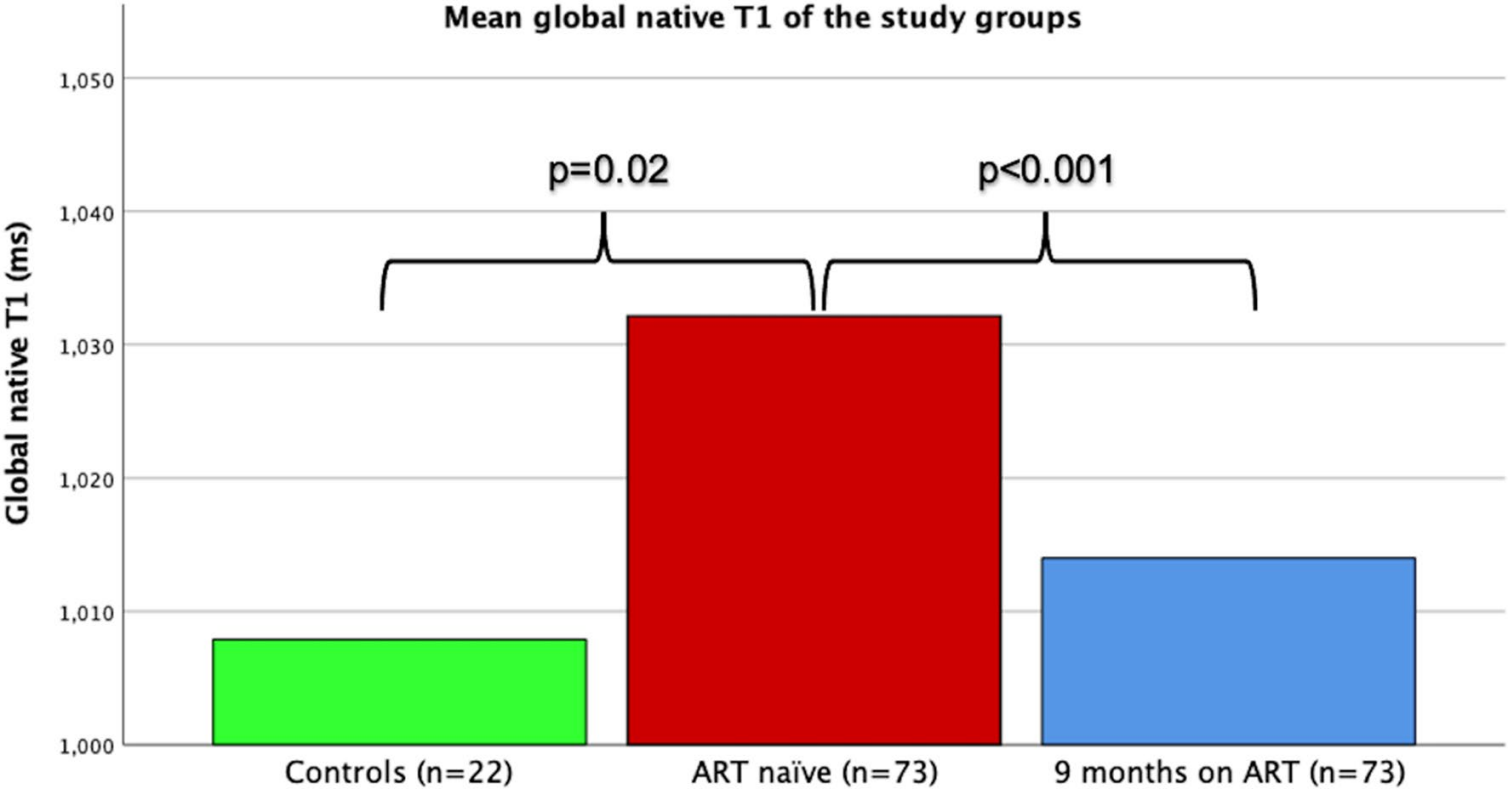
Bar graph demonstrating the mean global native T1 of the study groups. Compared with non-HIV controls, the ART naïve group demonstrated elevated global native T1. After the initiation of ART, the global native T1 of the HIV infected group decreased significantly and, although still relatively elevated, proved to be statistically similar to the non-HIV control group (1008 ± 31 vs 1014 ± 34, p = 0.5)

After ART initiation, significant changes in the HIV group’s mapping were observed. The global native T1 and ECV showed significant change towards normalisation and were statistically similar at 9 months when compared to the control group (Table 3 and Fig. 3). No significant change was observed in the global T2 after ART initiation.

### Late gadolinium enhancement

LGE was 5 times more prevalent in the HIV infected group compared with the control group (49% vs 10%, p = 0.02). Although unexpected, one control had LGE in keeping with non-ischaemic replacement fibrosis. In the HIV group, more than two thirds of visualised LGE was present in the basal segments and most frequently involved the inferoposterior walls. The pattern of LGE was predominantly subepicardial, with a smaller number of participants demonstrating midmyocardial enhancement, or a combination of the two. In all cases where LGE was visualised at baseline, LGE was seen at 9 months as well. Two cases were thought to have de-novo LGE at follow up. Three cases had LGE at follow up that did not meet study criteria of LGE at the initial evaluation.

### Persistent viraemia subgroup

Fifteen HIV infected persons demonstrated persistent viraemia and this group’s study parameters are shown in Table 4. All these cases had a history of poor compliance on ART preceding follow up. Although not proven to be significant, a marginal increase in pericardial effusion prevalence was observed over time (67% to 73%, p = 0.7).

**Table 4:** Subgroup analysis of persons with viral load > 200 copies/ml after the initiation on ART

Parameter	Persistent viraemia group	Persistent viraemia group	p value
	n = 15	n = 15	
	(Naïve)	(ART)	
CD4 count (cells/μl)	277 (214 to 355)	388 (209 to 529)	**0.009**
CD8 count (cells/μl)	980 (774 to 1536)	671 (465 to 1018)	**0.005**
HIV viral load (copies/ml)	77,005 (7478 to 350,082)	2389 (20 to 89,339)	0.7
HIV viral load (log copies/ml)	4.9 (3.9 to 5.5)	3.4 (1.3* to 5.0)	0.2
Presence of pericardial effusion	10 (67%)	11 (73%)	0.7
Global T2 SIR	1.5 ± 0.2	1.5 ± 0.2	0.5
Global native T1 mapping (ms)	1040 ± 27	1033 ± 39	0.3
Global native T2 mapping (ms)	48 ± 3	49 ± 3	0.4
Global ECV (%)	27 ± 4	26 ± 3	0.1
EGE	3.4 ± 1.1	3.3 ± 0.9	0.6
LGE present	7 (47%)	9 (60%)	0.7

Bold values indicate *p* value of ≤ 0.05

Continuous variables are mean ± standard deviation or median (interquartile range) unless otherwise specified

*LV* left ventricle, *RV* right ventricle, *SIR* Signal intensity ratio, *ECV* Extracellular volume, *LGE* late gadolinium enhancement

^*^20 copies/ml (log = 1.3) is the lower limit of measurement for our HIV-1 assay

The persistent viraemia group’s global native T1 did not decrease to the same degree as the greater HIV cohort and differences are evident when compared with the virally supressed participants (n = 58) that represent 79% of the HIV infected cohort (see Table 5). No significant difference in global native T1 was evident after 9 months on ART in the virally unsuppressed group (p = 0.3). The virally supressed subgroup demonstrated a threefold decrease in the global native T1 time compared with the persistent viraemia subgroup (the mean decrease in T1 of these two subgroups at recruitment and 9 months were 21 ms and 7 ms respectively). Furthermore, no significant difference in the global ECV of the persistent viraemia group was seen over time as was seen with virally supressed individuals. The global T2 in the virally unsuppressed subgroup increased by one ms, although this did not prove to be statistically significant.

**Table 5:** Subgroup analysis of virally supressed individuals (< 200 copies/ml after the initiation on ART)

Parameter	Virally supressed group	Virally supressed group	p value
	n = 58	n = 58	
	(Naïve)	(ART)	
CD4 count (cells/μl)	292 (152 to 432)	378 (274 to 633)	**< 0.001**
CD8 count (cells/μl)	789 (575 to 1044)	608 (456 to 821)	**< 0.001**
HIV viral load (copies/ml)	85,320 (11,604 to 298,008)	20* (20* to 22)	**< 0.001**
HIV viral load (log copies/ml)	4.9 (4.1 to 5.5)	1.3* (1.3* to 1.3*)	**< 0.001**
Presence of pericardial effusion	39 (67%)	35 (60%)	0.6
Global T2 SIR	1.5 ± 0.2	1.5 ± 0.2	0.5
Global native T1 mapping (ms)	1030 ± 48	1009 ± 31	**< 0.001**
Global native T2 mapping (ms)	48 ± 3	48 ± 2	0.2
Global ECV (%)	26 ± 4	24 ± 3	**0.006**
EGE	4.1 ± 2.8	3.7 ± 2.3	0.4
LGE present	28 (48%)	31 (53%)	0.5

Bold values indicate *p* value of ≤ 0.05

Continuous variables are mean ± standard deviation or median (interquartile range) unless otherwise specified

*LV* left ventricle, *RV* right ventricle, *SIR* Signal intensity ratio, *ECV* Extracellular volume, *LGE* late gadolinium enhancement

^*^20 copies/ml (log = 1.3) is the lower limit of measurement for our HIV-1 assay

### Bivariate analysis of parameter changes over time

#### Global native T1

The change in global native T1 showed a moderate positive correlation with the global change in ECV (r_s_ = 0.57, p < 0.001) and the change in hsCRP (r_s_ = 0.53, p < 0.001). In addition, a low positive correlation was seen with the change in global T2 (r_s_ = 0.43, p < 0.001) as well as the change in HIV viral load (log) (r_s_ = 0.39, p < 0.001). A low negative correlation with the change in CD4 count was seen (r_s_ = − 0.28, p < 0.02). No significant correlation with LVEF and LV end-diastolic volume (LVEDV) indexed to height were observed.

#### Global T2

The change in global T2 demonstrated a low positive correlation with the change in global ECV (r_s_ = 0.34, p = 0.004) and HIV viral load (log) (r_s_ = 0.30, p < 0.001). A low negative correlation was seen with the change in LVEF (r_s_ = − 0.31, p = 0.007).

#### Global ECV

The change in ECV showed a low positive correlation with the change in hsCRP (r_s_ = 0.33, p = 0.005).

### Multivariate analysis

**Fig. 4:**
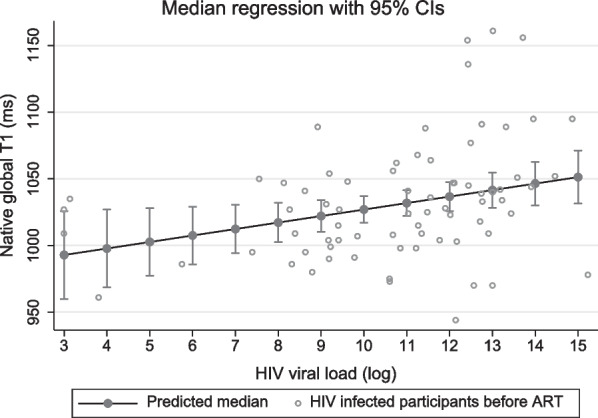
Median regression line (R^2^ = 0.2) for the outcome variable global native T1 with 95% confidence intervals (CI’s) for HIV infected participants at baseline (n = 73). The predicted median HIV viral load (log) is shown when the model is adjusted for age, sex, tobacco use, body mass index and CD4 count. The HIV viral load at the time of HIV diagnosis demonstrates a significant association with global native T1 (p = 0.02) with a coefficient of 5 (95% CI 1 to 9). This reflects an estimated median increase of 5 ms in the native global T1 for every log increase in the HIV viral load

The quantile regression model for global native T1 is shown in Fig. 4. The HIV viral load (log) was significantly associated with the global native T1 at the time of HIV diagnosis (p = 0.02) when adjusted for age, sex, tobacco, BMI, and CD4 count.

### Inter-reader reliability

The re-read of multiparametric tissue characteristics demonstrated good to excellent ICC between readers. The quantitative parameters were analysed, and their correlations included: Global native T1 (ICC = 0.85), Global T2 (ICC = 0.95), global T2 SIR (ICC = 0.86), EGE SIR (ICC = 0.82), and ECV (ICC = 0.98). Probability values were all calculated to be highly significant (p < 0.001). Excellent inter-rater reliability was shown for the identification of pericardial effusion (Kappa = 1.0, p < 0.001) and qualitative assessment of LGE (Kappa = 0.86, p < 0.001).

## Discussion

Our study is the first to prospectively evaluate the myocardial tissue characteristics of people living with HIV before and after initiation on ART. By acquiring quantitative, multiparametric mapping before and on ART, we were able to compare myocardial tissue characteristics and imaging biomarkers at different time points. Compared with currently available literature, our study better represents women and persons living in sub-Saharan Africa, the regions where more than two-thirds of the HIV burden is located [31]. Furthermore, our participants were young and essentially free from cardiovascular comorbidities, limiting additional confounding factors.

Our key findings were:

- At the time of HIV diagnosis, people living with HIV were found to have a high prevalence of pericardial effusion and globally increased native T1, T2 and ECV compared with controls. The global native T1 was significantly associated with the HIV viral load. These findings suggest subclinical myocardial oedema, likely due to HIV-associated myocardial inflammation.
- Global native T1 and ECV decreased on ART and were comparable to HIV uninfected persons at 9 months. The restitution of native T1 and ECV were associated with increased CD4 count, decreased HIV viral load and systemic inflammation, further supporting the inflammatory hypothesis.
- Global T2 was not seen to decrease at 9 months and may point to the presence of chronic myocardial oedema from inflammation, despite ART.
- A high prevalence of unchanging LGE was seen in people living with HIV and the pattern and distribution are in keeping with replacement fibrosis from non-ischaemic myocardial injury.

### Subclinical myocardial oedema

Myocardial fibrosis and/or oedema may explain the raised native T1 observed in our HIV infected group [10, 32]. Native T1 measurement allows for the uncontrasted assessment of a variety of pathological processes (ranging from subclinical to end-stage disease) in both the myocyte (intracellular compartment) and the interstitium (extracellular compartment) [33]. Although changes are not specific to a particular disease process, native T1 mapping is a sensitive tool to assess pathology related to oedema, necrosis, fibrosis, protein-, lipid-, and iron deposition [33] and is superior to LGE and T2-weighted imaging to detect myocardial inflammation [34, 35]. In addition to focal pathology, T1 mapping is able to provide information on diffuse myocardial disease and has distinct technical advantages over LGE imaging [36].

Notably, our mapping methodology included an endocardial and epicardial offset to increase both accuracy and reproducibility of measurements of the midmyocardium. Consequently, the subepicardial abnormalities visualised on LGE-imaging are not fully reflected in the native T1 measurements and these two parameters should be considered independently. In accordance with our study hypothesis, a decrease in the native T1 time was identified in the HIV infected group after initiation of ART. The notion that the native T1 decrease was due to the reversal of diffuse myocardial fibrosis is controversial and less likely here [37]. Our findings are better explained by the improvement of subclinical myocardial oedema secondary to ART initiation as the decrease in native T1 was associated with a decrease in HIV viral load, hsCRP, and an increase in CD4 count. Furthermore, the ventricles of our HIV infected cohort were heavier compared with controls, in keeping with observations from prior research [10, 21, 38]. Although not conclusive, we have demonstrated signal that the native T1 decrease in persons with suboptimal compliance and persistent viraemia do not show the same degree of native T1 decrease. These findings all support oedema as the cause for the native T1 findings in the study.

Increased signal in T2-weighted imaging is specific, although less sensitive, for detecting increased absolute, as well as free intracellular water in the myocardium [39]. The updated Lake Louise Criteria have incorporated T1- and T2 mapping abnormalities as main criteria to diagnose myocardial inflammation [35]. The presence of raised T2 mapping values as seen in our HIV infected cohort, coupled with high native T1 times and a high prevalence of pericardial effusion, provides further support for the presence of myocardial inflammation. The observation that the T2 mapping values did not decrease concurrently with T1 mapping, may be explained by the superior sensitivity of T1 to detect myocardial tissue abnormalities. As T2 mapping has excellent specificity to detect myocardial oedema, this provides evidence for the incomplete resolution of chronic myocardial oedema in the HIV group. This finding is in keeping with CMR research from a cohort of ART experienced people living with HIV, that demonstrated elevated T1 and T2 despite viral suppression [26].

Our ECV data is also in keeping with interstitial expansion secondary to extracellular oedema at the time of HIV diagnosis, with subsequent improvement on ART at 9 months. ECV has the ability to detect milder interstitial expansion not readily detectable by LGE and predicts clinical outcomes that has been shown to be as important as LVEF when estimating prognosis [40, 41]. The decrease in the ECV seen at 9 months in our data is likely indicative of improved cardiovascular risk for PLWH, but will require formal evaluation in outcome studies.

### Myocardial fibrosis

**Fig. 5:**
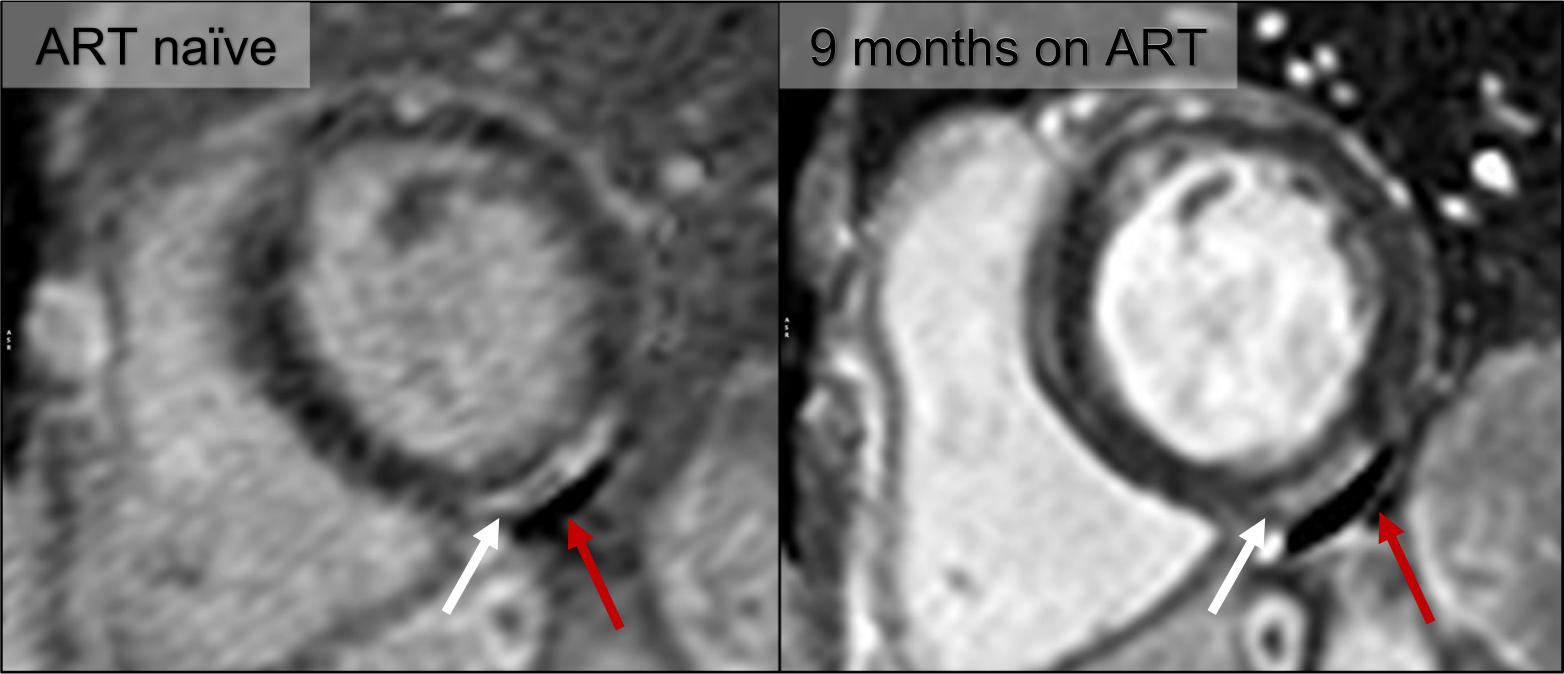
Basal short axis, phase sensitive inversion recovery late gadolinium enhancement (LGE) image of an HIV infection participant before and on ART. Bright, subepicardial LGE (white arrows) is present in the basal inferior wall and does not resolve on ART. Note the non-resolving pericardial effusion (red arrows)

The focal LGE in our cohort is in keeping with non-ischaemic, replacement type fibrosis (Fig. 5). In accordance with the largest LGE study in people living with HIV to date, the prevalence of fibrosis in our cohort was significantly higher in the HIV infected group compared with controls [42]. LGE did not change significantly over time, further supporting the presence of myocardial fibrosis. Comparing our LGE findings with published data in ART naïve persons from a low- to middle income country, the prevalence and pattern of LGE do not differ significantly (49% vs 39% between studies with predominantly subepicardial and midmyocardial LGE reported) [12]. A study from a high income country has reported LGE in as many as 85% of ART naïve individuals, although this study only evaluated 13 ART naïve persons [10].

Our findings support the growing body of evidence that, in addition to myocardial oedema, a component of replacement-type, myocardial fibrosis may be present in people living with HIV [10, 12]. The long- term significance of this finding, already demonstrable at the time of HIV diagnosis, is unknown. Although diffuse myocardial fibrosis have been linked to poor outcomes in persons with heart failure [43], the long term implications of fibrosis seen in people living with HIV require further study. An autopsy study that evaluated HIV infected persons with sudden cardiac death found that total fibrosis (interstitial-, perivascular-, and replacement fibrosis) was on average 72% higher in people living with HIV compared to HIV uninfected persons [32]. Although a higher rate of sudden cardiac death attributable to arrythmia was not conclusively shown, the reported incidence rate ratio of 1.87 suggests this is possible. Longitudinal studies evaluating the influence of different types of myocardial fibrosis on prognosis are lacking.

### ART and CVD

ART itself may have deleterious effects on cardiovascular health and is another mechanism of CVD in people living with HIV. Our cohort was on a dolutegravir-based ART regimen and unfortunately little is known on the long term cardiovascular effects of integrase inhibitors. Integrase inhibitors are thought to be have a more favourable cardiovascular side effect profile compared with protease inhibitors that have well-described pro-atherogenic effects [44]. However, a recent publication has shown a possible link with myocardial infarction, stroke, and cardiovascular intervention [45]. ART side effects with a co-existing subclinical cardiomyopathy remains unstudied. Our study explored inadequate viral suppression as a mechanism for CVD and not specifically the type of ART, as most of our participants received the same regimen. Therefore our results cannot necessarily be extrapolated to all ART regimens.

## Limitations

Our study has some important limitations. Although a decrease in the global native T1 suggests improvement of oedema, it is unclear how much residual oedema is present as the global T2 was not seen to decrease. Findings from persons already on ART, suggest subclinical myocardial inflammation persists, despite ART. Although the global native T1 of our HIV group improved, it remained 6ms higher than controls. A larger prospective sample size would be required to comment on residual oedema.

In the presence of global myocardial oedema, we are unable to comment on the presence and extent of diffuse interstitial fibrosis, as global native T1 and ECV may be raised in both these pathologies. LGE is considered the gold standard for diagnosing myocardial scar [46] and was utilised in our study as a qualitative, rather than a quantitative parameter. In the presence of global pathology, LGE is a less sensitive diagnostic tool, as no normal reference myocardium may be present. Although semi-automated, quantitative LGE assessment is improving and shows promise, consensus on quantification-methodology has not been reached. Current techniques show considerable difference across cardiac pathologies [47]. Notably, the LGE quantification techniques remain subjective, requires considerable human input and interpretation is still required to distinguish true signal from artefact [46]. The most reproducible of these methods, appears to be the full width-half max method which has demonstrated similar estimations of LGE volume % compared to manual contouring [47].

The CMR studies were analysed unblinded and there is risk of potential bias. This risk was mitigated by utilising pre-specified measurement and reporting methodology as described earlier. Furthermore, the analysis of a random sample of cases by a blinded reader demonstrated excellent correlation of quantitative as well as qualitative parameters between the two readers. This supports a high level of reproducibility in our data. However, the absence of a blinded analysis remains a significant limitation and the possible over-estimation of abnormalities cannot be fully eliminated.

The explorative persistent viraemia subgroup is small. A higher number of participants would allow for more reliable comparison with virally supressed individuals. However, this group was included to assist with hypothesis generation and provide unique and novel data.

## Conclusion

Our data suggest that a combination of subclinical myocardial oedema and fibrosis are present in people living with HIV. This was already demonstrable at the time of HIV diagnosis and may represent subclinical HIVAC. ART initiation with consequent viral suppression, increased CD4 count, and decreased systemic inflammation have overall beneficial effects, as the markers of myocardial oedema were seen to decrease over the study term. However, a component of chronic myocardial oedema remains a possibility. Myocardial scar, as demonstrated by LGE, remained unchanged during the study. Furthermore, systolic function did not improve on ART, providing additional evidence that a component of non-reversable myocardial injury is present. It is not known whether the underlying pathological mechanisms involved have been halted by ART or merely slowed. Mid- to long term prospective studies are required to evaluate subtle changes over time and provide additional information on the prognostic implications of subclinical myocardial fibrosis.

## Data Availability

The datasets of the study are not publicly available but are available from the corresponding author and with permission from Stellenbosch University upon reasonable request.

## Abbreviations

6MWT: 6 minute walk test
ART: Antiretroviral therapy
BMI: Body mass index
bSSFP: Balanced steady state free precession
CMR: Cardiovascular magnetic resonance
CVD: Cardiovascular disease
ECG: Electrocardiogram
ECV: Extracellular volume fraction
EGE: Early gadolinium enhancement
eGFR: Estimated glomerular filtration rate
HIV: Human immunodeficiency virus
HIVAC: HIV-associated cardiomyopathy
hsCRP: High sensitivity C-reactive protein
ICC: Interclass correlation coefficient
LGE: Late gadolinium enhancement
LV: Left ventricle/left ventricular
LVEDV: Left ventricular end-diastolic volume
LVEF: Left ventricular ejection fraction
MOLLI: Modified Look Locker inversion recovery
PCR: Polymerase chain reaction
ROI: Region-of-interest
RV: Right ventricle/right ventricular
SARS CoV-2: Severe acute respiratory syndrome coronavirus 2
STIR: Short-tau inversion recovery
TB: Tuberculosis
WHO: World Health Organisation
